# BOLD fMRI at 9.4T with 3D stack-of-spirals readouts

**DOI:** 10.1007/s10334-025-01298-4

**Published:** 2025-09-29

**Authors:** Alejandro Monreal-Madrigal, Denizhan Kurban, Desmond H. Y. Tse, Dimo Ivanov, Nicolas Boulant, Benedikt A. Poser

**Affiliations:** 1 Maastricht Brain Imaging Centre, Faculty of Psychology and Neuroscience, Maastricht University, Maastricht, The Netherlands; 2 Scannexus BV, Maastricht, The Netherlands; 3 University Paris-Saclay, CEA, CNRS, BAOBAB, NeuroSpin, Gif-Sur-Yvette, France

**Keywords:** BOLD fMRI, Ultra High Field, Non-Cartesian readouts, Pulseq, Open-source

## Abstract

**Objective:**

To investigate the use of spiral readouts for sub-millimeter BOLD fMRI at 9.4T and to verify simulations of the BOLD point spread function (PSF) with functional experiments.

**Materials and Methods:**

Spiral readouts were evaluated through simulations and functional experiments to test their performance in sub-millimeter BOLD fMRI. Both spiral-out and spiral-in readout strategies were considered, with attention to echo time (TE) relative to T_2_*.

**Results:**

We confirmed that a TE shorter than T_2_* can be employed for spiral-out readouts without compromising BOLD sensitivity. The use of shorter TE provided a reduced repetition time (TR), improved temporal signal-to-noise ratio (tSNR), and minimized off-resonance effects.

**Discussion:**

Spiral-in readouts were found to be mainly useful for lower-resolution applications at ultra-high field (UHF). In contrast, segmented spiral-out readouts demonstrated strong potential for mesoscopic BOLD fMRI at ultra-high fields (> 7T).

**Supplementary Information:**

The online version contains supplementary material available at 10.1007/s10334-025-01298-4.

## Introduction

Functional MRI (fMRI) is an MRI modality that non-invasively measures brain activity. It was first introduced as blood oxygenation level dependent (BOLD) [1] contrast, following the observation that changes in blood deoxyhemoglobin content during neuronal activation alter the MRI signal. Typical spatial and temporal resolutions for human fMRI experiments have been on the order of a couple of millimeters and seconds, respectively.

The desire for higher resolutions in fMRI has been one of the main drivers of ultra-high field MRI [2–4]; increasing the field strength provides a nearly quadratic increase in SNR [5]. Gradient coils with higher gradient amplitudes and slew rates are also being developed [6, 7], providing significant speed-up in echo-planar and spiral readouts. In addition, receive coils with high channel count RF receive arrays [8] are being developed, allowing higher parallel acceleration by k-space undersampling. These hardware advances, together with new sequence developments, have made it possible to achieve sub-millimeter resolutions and sub-second acquisitions. These tools can be used by neuroscientists to explore the human brain at high temporal and spatial resolutions with unprecedented detail.

BOLD fMRI is an indirect measure of neuronal activation; it measures the hemodynamic response function (HRF) which is a combination of different physiological processes: cerebral blood flow (CBF), cerebral blood volume (CBV), and CMRO_2_ (oxygen consumption) [9]. Non-BOLD contrasts have been proposed to measure those effects more directly, such as calibrated fMRI to measure CMRO_2_ [10, 11], arterial spin labeling (ASL) [12, 13] for CBF, and vascular space occupancy (VASO) [14, 15] for CBV. At ultra-high field (UHF), however, these pose different implementation challenges; for example, ASL and VASO make use of RF preparation modules that increase the minimum achievable repetition time (TR) and require a high use of RF power for the saturation and inversion pulses [2]. For these reasons, BOLD contrast is still the most widely used in fMRI studies, also at UHF.

In functional imaging, small changes in T_2_*, caused by the local change in deoxyhemoglobin concentration that accompanies neuronal activation, manifest and thus the highest BOLD signal difference between activation and rest is observed when TE ~ T_2_* of the gray matter [16]. The most commonly used readout strategy for BOLD fMRI is Cartesian echo-planar imaging (EPI) [17], which achieves high sampling efficiency and enables volume repetition times on the order of seconds at the typically desired resolutions for fMRI applications. In the past decade, the incorporation of CAIPIRINHA [18] acceleration into 2D simultaneous multi-slice [19–21] and 3D EPI [22–24] has improved EPI and further increased its neuroscientific use.

Alternatives to EPI for fast imaging are non-Cartesian readouts, of which the most common are spiral and radial [25, 26]. Other non-Cartesian readouts that have been proposed for fMRI can be found in Refs. [27–29]. Spiral readouts with full k-space sampling were introduced for BOLD fMRI at 3T [30–32]. Following advances in spiral reconstruction and off-resonance correction [33, 34], spiral imaging has been revisited at 7T by Engel et al. [35] and for 2D BOLD fMRI by Kasper et al. [36]. Recently, spiral readouts have also been used for balanced SSFP BOLD in work by Valsala et al. [37], in which the authors exploited the flexibility to choose TE to improve the functional contrast. Spirals have also been used for different non-BOLD contrasts such as ASL [38] and VASO [39], where a short echo time is desired to improve the inherent low SNR of these techniques. Since these types of readouts do not fall into a Cartesian grid, the use of the FAST Fourier Transform is not possible; the reconstruction is then typically performed offline and it is a computationally expensive process.

The simplest design of a spiral readout follows an Archimedean spiral. In this case, an entire k-space plane (2D) is acquired after an excitation (single-shot). Each plane can also be acquired using more excitations (multi-shot) which shortens each readout and reduces vulnerability to *B*_0_ off-resonance, however, at the cost of increased volume TR. The k-space can be transversed from the center to the edge (spiral-out) or starting from the edge and moving inwards (spiral-in). A combination of both (spiral-out-in) acquires two echoes per excitation. The center of the k-space can be sampled more densely than the outer part (variable density spirals). Stacking spiral planes in the partition direction produce a 3D stack-of-spirals pattern. To improve the undersampling behavior, k-space planes can be rotated [40]. All these parameters affect the contrast, acquisition duration, and overall image quality, and careful selection of them is important for a successful implementation of spiral imaging.

The widespread use of spiral readouts has been limited due to practical challenges in their implementation. One of them has been the need to account for trajectory deviations as well as eddy currents, which requires knowledge of the trajectory executed by the scanner. The trajectory can be measured with imaging techniques [41], by use of special hardware such as field cameras [34], or measurement of the gradient impulse response function [42] which can subsequently be applied to predict the achieved gradient waveform. Another challenge is the reconstruction process, which typically makes use of the computationally demanding non-uniform Fourier transform (NUFFT) [43], and hence often impractical to implement on the scanner’s own image reconstruction hardware. Finally, *B*_0_ off-resonance results in spiral image blurring with a 2D point spread that is much harder to correct for than the 1D phase-encoding distortion in EPI, which can be addressed with straightforward post-processing (e.g., FSL top-up [44]). The continuously changing phase-encoding direction and rate of k-space traversal in spirals cause a complex phase accrual and blurring that need to be addressed during the image reconstruction [45], considerably adding extra computational demands and reconstruction times.

To avoid off-resonance effects due to *B*_0_ field inhomogeneities when using long spiral readouts, an off-resonance frequency term needs to be included in the SENSE signal model [46]:

1 $${S}_{\gamma }(t)={\int }_{V}{c}_{\gamma }(r)m(r){e}^{-ik(t)r}{e}^{-i\Delta {\omega }_{0}(r)t}dV$$

where *s*(*t*) is the k-space data from each receiver channel *ɣ*, *c*(*r*) is the complex spatial sensitivity of the *ɣ* coil, *m*(*r*) is the magnetization, *k*(*t*) is the k-space trajectory, and the term Δ*ω*_0_ is the angular off-resonance frequency, proportional to field inhomogeneities. Different methods for off-resonance correction exist, such as time segmented [47], multi-frequency interpolation [48], and gridding approaches [49]. Equation 1 can be discretized and a linear system of equations can be created with the k-space data:

2 $$s=Em,E={c}_{\gamma }(r){e}^{-ik(t)r}{e}^{-i\Delta {\omega }_{0}(r)t}$$

This system of equations can be solved iteratively with a regularized least-squares cost function:

3 $$\widehat{m}=\mathrm{argmin}\frac{1}{2}||Em-s|{|}_{2}^{2}+\frac{\beta }{2}R(m),$$

where *β* is the regularization parameter and *R*(*m*) is a regularization function that is used to ensure that the algorithm converges to a stable solution, common regularization functions are *L*_1_ norm, *L*_2_ norm and total variation (TV) [50]. Different algorithms exist to solve Eq. 3, some of the most widely used are CG-SENSE [33], ADMM [51], and FISTA [52].

In BOLD fMRI, the highest contrast between activation and rest is observed at a TE approximately equal to T_2_*. Engel et al. [53] proposed the concept of the BOLD point spread function (PSF) to better characterize the impact of the readout and TE on BOLD functional experiments. Using simulations, they showed that a TE ~ T_2_* achieves the highest BOLD sensitivity with spiral readouts.

In MR Fourier encoding, the point spread function (PSF) is the inverse Fourier of the k-space filter *H*(*k*):

4 $${\mathrm{PSF}}\left( r \right) = F^{ - 1} \left( {H\left( k \right)} \right)$$

with $k=[{k}_{x},{k}_{y},{k}_{z}{]}^{T}$. The k-space filter *H*(*k*) comprises the sampling of k-space at discrete points on a finite support and a weighting function:

5 $$H(k)={H}_{\mathrm{sampling}}(k)\cdot {H}_{\mathrm{weight}}(k)$$

In gradient echo experiments, a mono-exponential signal decay weighting ${H}_{\mathrm{weight}}^{{T}_{2}*}$ is imposed. In a BOLD experiment, the measure of interest is the small differences arising from temporal changes in T_2_*. The BOLD weight filter ${H}_{\mathrm{weight}}^{\mathrm{BOLD}}$ is the derivative of the T_2_* weight filter:

6 $${H}_{\mathrm{weight}}^{{T}_{2}*}(k)={\mathrm{exp}}^{-t(k)/{T}_{2}*} , {H}_{\mathrm{weight}}^{\mathrm{BOLD}}(k)=\frac{\partial {H}_{\mathrm{weight}}^{{T}_{2}*}(k)}{\partial {T}_{2}*}=\frac{t(k)}{{{T}_{2}^{*}}^{2}}{ \mathrm{exp}}^{-t(k)/{T}_{2}*}$$

BOLD imaging is then characterized by a differential PSF:

$${\mathrm{PSF}}_{\mathrm{BOLD}}(x,{T}_{2}^{*},\Delta {T}_{2}^{*})={\mathrm{PSF}}_{\mathrm{GE}}(x,{T}_{2}*+\Delta {T}_{2}^{*})-{\mathrm{PSF}}_{\mathrm{GE}}(x,{T}_{2}*)=\frac{\partial {\mathrm{PSF}}_{\mathrm{GE}}(x,{T}_{2}*)}{\partial {T}_{2}*}\Delta {T}_{2}^{*}$$

7 $$={F}^{-1}({H}_{\mathrm{sampling}}{H}_{\mathrm{weight}}^{\mathrm{BOLD}})\Delta {T}_{2}^{*}={F}^{-1}({H}_{\mathrm{sampling}}(k)\frac{t(k)}{{{T}_{2}^{*}}^{2}}{ \mathrm{exp}}^{-{\boldsymbol{t}}({\boldsymbol{k}})/{T}_{2}*})\Delta {T}_{2}^{*}$$

In this work, we combine some of the previously mentioned hardware and sequence advances. We use a 3D stack-of-spiral sequence for BOLD fMRI on a 9.4T scanner, equipped with high-performance head-only gradients for more efficient k-space coverage. We experimentally verify the BOLD point spread function simulations by Engel et al. [53] for spiral readouts and further investigate the benefits of spiral readouts for sub-millimeter fMRI at UHF. We also provide the complete pipeline for sequence, reconstruction, and analysis for spiral fMRI data.

The goal of this project was to better characterize the tradeoffs of spiral BOLD at laminar resolution and gain insights into the challenges when aiming for yet higher resolutions (<0.6 mm) fMRI and field strengths (>9.4T) as envisaged by the AROMA project. With further shortening of T_2_* and increases in *B*_0_ inhomogeneities, single-shot EPI will also become increasingly challenging at high resolution, with the prohibitively long readout precluding suitably short TE, unless using segmentation, which comes at TR and tSNR penalties.

## Methods

Simulations of the BOLD point spread function were performed for a range of T_2_* values (T_2_* = 20–26 ms) to determine a suitable TE for 0.6 mm isotropic-resolution BOLD fMRI with dual-shot spiral-out (DS-SO) and dual-shot spiral-in (DS-SI) readouts. In previous work, we concluded that high-resolution single-shot spiral-out images at 9.4T suffer severe off-resonance effects and are not usable [54]; for this reason, we focus on dual-shot spiral readouts in this work. Echo times ranging from 2 to 30 ms were used for the spiral-out simulations, and 30 to 50 ms for spiral-in.

**Fig. 1:**
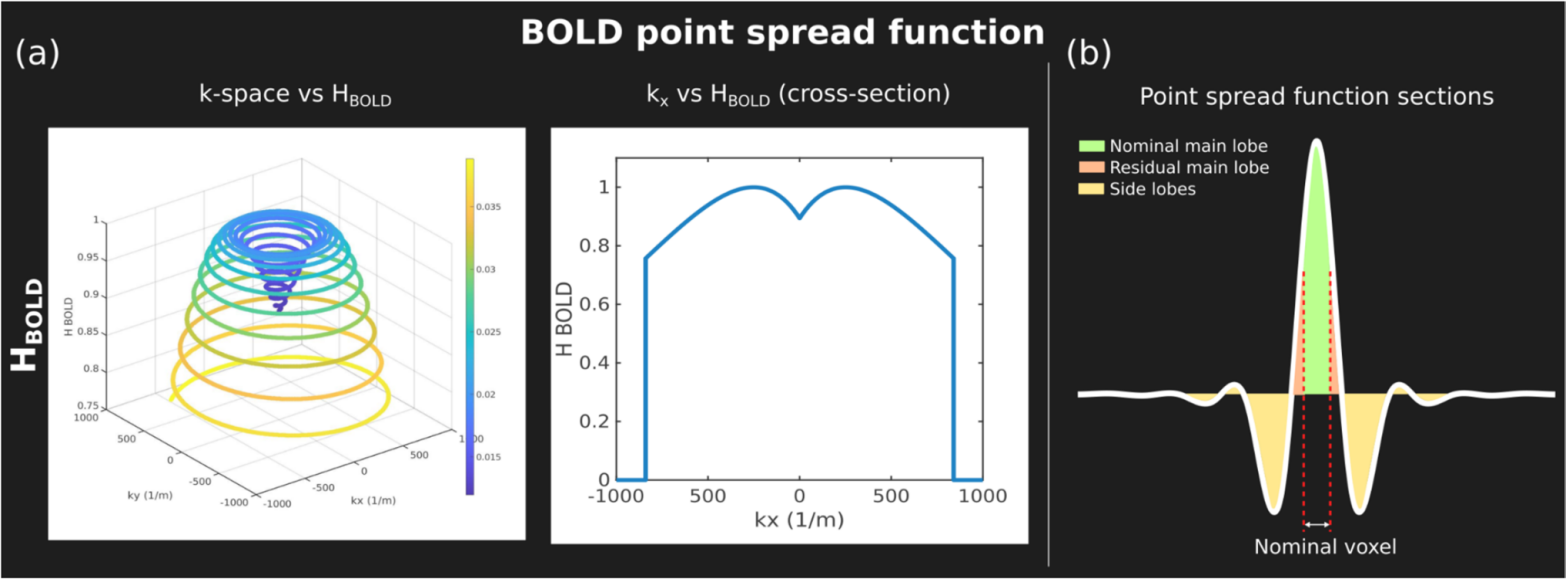
BOLD imaging filter (*H*_BOLD_) schematic depiction and main components of the BOLD point spread function from a spiral-out readout. **a** k-space vs *H*_BOLD_ and cross-section. **b** Real part of the BOLD PSF and its sections. The main lobe is divided into nominal main lobe (nominal voxel width) and residual main lobe. Side lobes include both the negative and positive ones

The real part of the PSF was divided in three different sections: nominal main lobe, residual main lobe, and side lobes (Fig. 1b), which were used to characterize each PSF similarly to Refs. [53, 55]. *BOLD resolution* (FWHM of main lobe), *sensitivity* (integral over the nominal main lobe), and *specificity* (relation of the integral over nominal main lobe and the integral of the absolute value of the residual main lobe and side lobes) were used as metrics:

8 $${\mathrm{res}}_{\mathrm{BOLD}}={\mathrm{FWHM}}_{\mathrm{PSF}},\hspace{0.25em}\hspace{0.25em}\mathrm{sensitivity}=\sum \mathrm{nom}.\text{ main lobe},\hspace{0.25em}\hspace{0.25em}\mathrm{specificity}=\frac{\sum \mathrm{nom}.\text{ main lobe}}{\sum |\text{side lobes}| +\sum | \mathrm{res}.\text{ main lobe}{|}}$$

Signal-to-noise ratio estimates were also obtained; the SNR of an image is proportional to:

9 $$\mathrm{SNR} \propto xyz\sqrt{{n}_{\mathrm{shots}}{T}_{\mathrm{acq}}} f(\mathrm{TR},\mathrm{TE})$$

10 $$f\left(\mathrm{TR},\mathrm{TE}\right)\propto \frac{\left(1-E\right)\mathrm{sin}\left(\alpha \right)}{1-\mathrm{cos}\left(\alpha \right)E}{e}^{\frac{-\mathrm{TE}}{{T}_{2}^{*}}},E={e}^{\frac{-\mathrm{TR}}{{T}_{1}}}$$

where *x*, *y*, *z* are the voxel dimensions, *n*_shots_ is the number of interleaves (lines or segments per plane), *T*_acq_ is the duration of the acquisition readout, and *f*(TR,TE) is a function describing the dependence on the relaxation characteristics of the tissue for gradient echo.

Data were acquired on a Siemens 9.4T scanner equipped with a 16Tx-31Rx head coil [56] and an AC-84II head-only gradient with 80 mT/m peak amplitude and 330 T/m/s slew rate. When using spiral readouts covering a circular k-space, an area similar to that of a square k-space should be used to achieve the desired resolution. In this work, we used a k-space radius which is 1.13 times larger than the nominal one as proposed in Ref. [57]. To do so, an in-plane res_nom_ = 0.54 × 0.54 × 0.60 mm^3^ was used, resulting in an effective resolution res_eff_ = 0.6 mm isotropic. The spiral readouts were designed with a variable density *α* = 1.3, in-plane undersampling *R*_xy_ = 3.3, and a 120° rotation of segments between planes. An optimal gradient design algorithm [58] was used to achieve the desired k-space trajectory in the shortest time with *G*_max_ = 50 mT/m, SR_max_ = 250 T/m/s and BW = 312 kHz.

A total of nine healthy volunteers were scanned after providing written consent following the protocols of the local ethics committee. Four volunteers were scanned to validate the BOLD point spread function simulations. Five volunteers were later scanned to assess the relative utility of spiral-in and spiral-out readouts. To address the B_1_+ inhomogeneities in the visual cortex, we used pre-calculated RF shim settings (phase-only), previously obtained as an average of several subjects.

**Fig. 2:**
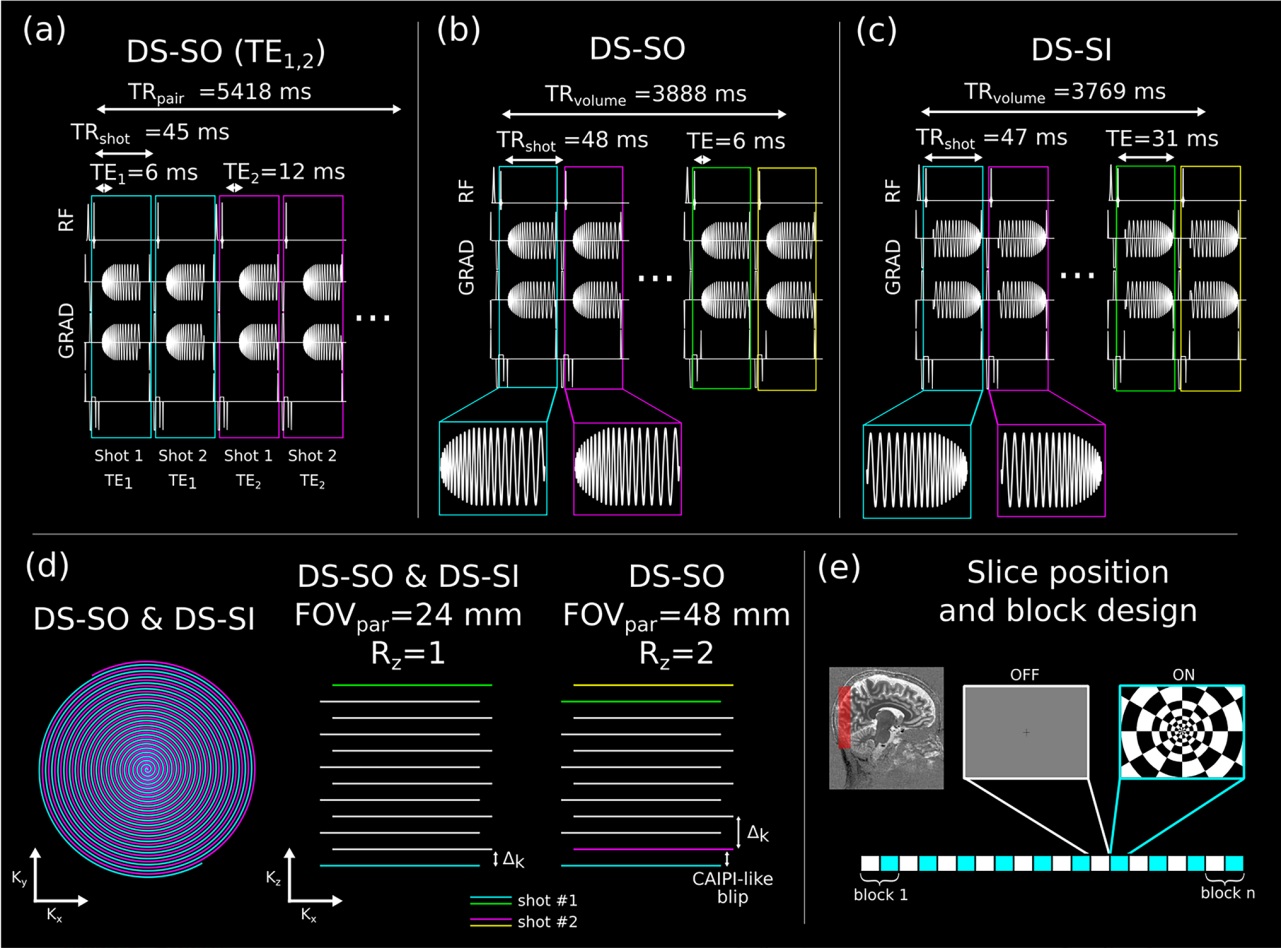
Schematic depiction of sequences, k-space trajectory, stimuli, and slice positioning. **a** Sequence used for the BOLD PSF experiments. The partitions of both TEs are acquired interleaved (e.g., TE_1_ (blue): 1st shot, 1st partition → TE_1_ (blue): 2nd shot, 1st partition → TE_2_ (purple): 1st shot, 1st partition → TE_2_ (purple): 2nd shot, 1st partition, etc.). A fat saturation module is used before the first excitation of each partition. The DS-SO (**b**) and DS-SI (**c**) sequence used for the spiral-in and spiral-out experiments. A fat saturation module is used before each excitation. **d** Schematic depiction of the k-space trajectories used in this work, for both the FOV_partition_ = 24 mm (*R*_*z*_ = 1) and the FOV_partition_ = 48 mm (*R*_*z*_ = 2); for the latter, a blip is applied after each shot to achieve CAIPIRINHA-like k-space coverage in the partition (kz) direction. **e** FOV orientation and paradigm block design using a flicker checkerboard

To validate the BOLD PSF simulations, a sequence with a dual-shot spiral-out (DS-SO) stack-of-spirals readout and two different nominal echo times (6 and 12 ms) was implemented in Pulseq [59]. The echo time of 6 ms was selected as a good compromise between the different quality metrics and the 12 ms one since simulations suggest it gives the highest sensitivity at T_2_* = 22 ms, the expected value of GM at 9.4T [60]. Budde et al. [61] reported an average GM T_2_* of 28.3 ± 6.8 ms at 9.4T, obtained from ME-GRE scans with 0.35 × 0.35 × 2.00 mm^3^ resolution. Plane partitions were interleaved between echo times, resulting in effectively simultaneous acquisition of the fMRI BOLD response at the two echo times. Fat saturation was applied before each excitation. The sequence furthermore contained a 1 ms long navigator module consisting of two 0.3 ms non-phase encoded FID 0.4 ms apart, applied at TE = 2 ms. The following parameters were used: FOV = 140 × 140 × 18 mm^3^, 30 *k*_*z*_ partitions with linear encoding order, TE_1_/TE_2_ 6/12 ms, TR_shot_ = 45 ms, TR_volume_ = 2.7 s, TR_pair_ = 5.41 s, FA = 15° and BWTP = 25. This resulted in a readout duration of 27 ms per shot. The sequence used RF spoiling with quadratic phase increments and gradient spoiling in three axes. A schematic depiction of this sequence can be found in Fig. 2a.

Seven 15 min functional runs were acquired (three volunteers underwent 2 runs, and another one 1 run). A full-screen flickering (approx. 8 Hz) black and white radial checkerboard programmed in Psychopy [62] was used as stimuli. Each block consisted of 7 TR_pair_ of rest (38 s) followed by 7 TRs of activation (38 s), effectively acquiring 14 volumes in each block (7 per echo time); each run consisted of 154 volumes per TE. The block design, stimuli, and slice positioning can be found in Fig. 2e.

To further investigate the performance of different spiral readouts, three volunteers were scanned with a dual-shot spiral-out (DS-SO) and dual-shot spiral-in (DS-SI) sequences. Most of the parameters were the same as described above, except for FOV = 140 × 140 × 24 mm^3^ and 40 *k*_*z*_ partitions. The DS-SO had a TE = 6 ms, TR_shot_ = 48 ms, TR_volume_ = 3.88 s, FA = 11° and a readout duration of 27 ms (per shot), whereas the DS-SI had a TE = 31 ms, TR_shot_ = 47 ms, TR_volume_ = 3.76 s, FA = 11°, and a readout duration of 27 ms (per shot). The TE = 6 ms ultimately was chosen following the simulations, which show that it is a reasonable compromise between the different metrics used in this work.

To test the acquisition of a larger FOV, two additional volunteers were scanned with the DS-SO sequence as described, and in addition with doubled FOV_partition_ (48 mm instead of 24 mm), but with *R*_*xy*_ = 3, *R*_*z*_ = 2 to achieve a similar TR and 8% phase oversampling to avoid foldover in the center slices; all other parameters were kept constant. Since a coronal view provides limited sensitivity changes in the AP direction, a kz blip was applied between shots to improve the aliasing pattern in the partition direction. This resulted in a CAIPIRINHA-like [18] pattern with shift Δ = 1 along kz, effectively resulting in an undersampling of *R*_*xy*_ = 6 and *R*_*z*_ = 1. A schematic depiction of these sequences and k-space trajectories can be found in Fig. 2b,c,d.

Each functional run lasted approximately 12 min, with the block design consisting of 8 TRs of rest followed by 8 TRs of activation. For DS-SO and DS-SI, this resulted in 31 s and 30 s block duration, respectively, and 192 volumes were acquired for each run. The same full-screen flickering checkerboard was used for the BOLD PSF experiments.

A 3D low-resolution fully sampled multi-echo GRE acquisition with segmented spiral-out readout was acquired before each functional run and used to calculate sensitivity and *B*_0_ maps. This sequence was FOV matched to the fMRI scans and used the following parameters: res_nom_ = 1.1 × 1.1 × 0.6 mm^3^, TE_1_/TE_2_/TE_3_ = 2.3/6.5/10.7 ms, TR = 62 ms (for SAR restrictions), FA = 11°, and BWTP = 25. Spiral parameters: 32 shots, *R*_*xyz*_ = 1, variable density *α* = 1, resulting in a readout duration of 3.2 ms (per shot) and total scan duration of 1.5 and 3 min for the 24 mm and 48 mm partition FOV, respectively.

Image reconstruction was performed offline using a pipeline based on the one presented in Ref. [39], using Neurodesk [63]. The first echo of the low-resolution ME-GRE scan was used to calculate the sensitivity maps using the ESPiRIT [64] implementation in MRIReco.jl [65]. These maps were then compressed to 16 virtual channels [66]. The three echoes were used to calculate the off-resonance map using the regularized field map estimation [67] as implemented in MRIFieldmaps.jl.

The GIRF predicted trajectory and *k*_0_ modulations were estimated from the nominal trajectory using previously acquired gradient impulse response function (GIRF) data following the method described in [68]. Dynamic off-resonance correction in k-space (DORK) [69] was applied to the raw data to correct for scanner drift. The phase of the first FID navigator of each volume was compared to the second one of the fMRI time series, and any phase difference was removed from the raw data. The DORK corrected functional raw data were merged with the GIRF predicted trajectory into an MRD file [70].

**Fig. 3:**
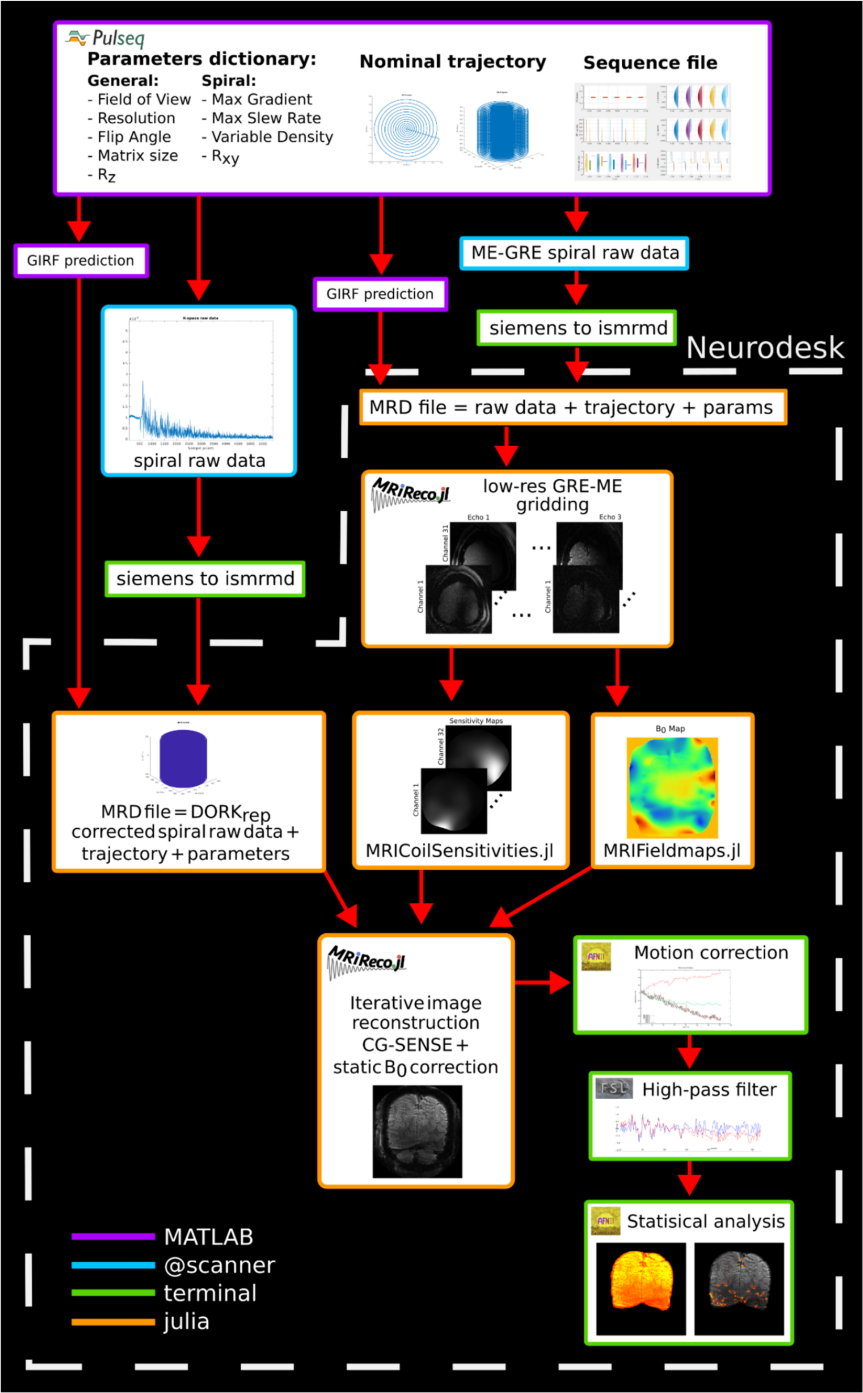
Overview of the 3D stack-of-spirals acquisitions, reconstruction, and analysis pipeline. *Sequence*: the Pulseq sequences are generated in MATLAB; nominal trajectories and parameter dictionaries are saved. The nominal trajectories are used to obtain the GIRF predicted ones. Fully sampled, highly segmented low-resolution GRE-ME and spiral functional data are acquired; both data sets are converted into MRD files. *Reconstruction*: The DORK-corrected spiral raw data is merged with the GIRF predicted trajectory and other parameters into an MRD file. Sensitivity and *B*_0_ maps are calculated from the low-resolution ME-GRE scan. The MRD file, sensitivity, and *B*_0_ maps are the input to the reconstruction in MRIReco.jl. *Analysis*: Rigid motion correction is performed with AFNI, high-pass filter in FSL, and the statistical analysis is done with AFNI. The reconstruction and analysis are done in Neurodesk

The sensitivity maps, off-resonance maps, and MRD file were the input to the reconstruction performed in MRIReco.jl using an ADMM solver and “*L*_1_” regularization, including a density compensation function [71] and multi-frequency interpolation *B*_0_ correction [48]. The number of iterations and stopping criteria was set to 30 and 1.2 × 10^–7^, respectively. The number of bins was selected as $\frac{4\Delta {\omega }_{\mathrm{max}}T}{\pi }$, where ${\Delta {\omega }_{\mathrm{max}}}$ is the maximum (absolute) off-resonance frequency and $T$ is the readout duration, resulting in 16 bins for the 27 ms long readout. Reconstruction was performed on a dedicated server running Ubuntu 22.04, with 1 TB of memory and two Intel Xeon Scalable Processor “Skylake” Gold 6140 with 18 cores each, for a total of 36 cores. Figure 3 shows an overview of the acquisition, reconstruction, and analysis pipeline.

The data analysis pipeline was implemented in Neurodesk [63] and consisted of: (1) motion correction using AFNI’s 3dAllineate command [72], the time series was registered to the second volume of the acquisition. (2) High-pass temporal filter using FSL with an FWHM of the duration of rest/activity blocks. (3) Mean time series and tSNR were computed as quality metrics using AFNI. (4) Activation maps were computed using a general linear model (GLM) and clustered using AFNI’s 3dDeconvolve and 3dclust commands. BOLD sensitivity in terms of BOLD contrast to temporal noise ratio (tCNR) was calculated for each voxel average block as tCNR = Δ*s*/*σ*_*n*_, with Δ*s* = mean BOLD signal change (mean activation − mean rest) and *σ*_*n*_ = standard deviation of GLM residual. No spatial smoothing was applied.

## Results

**Fig. 4:**
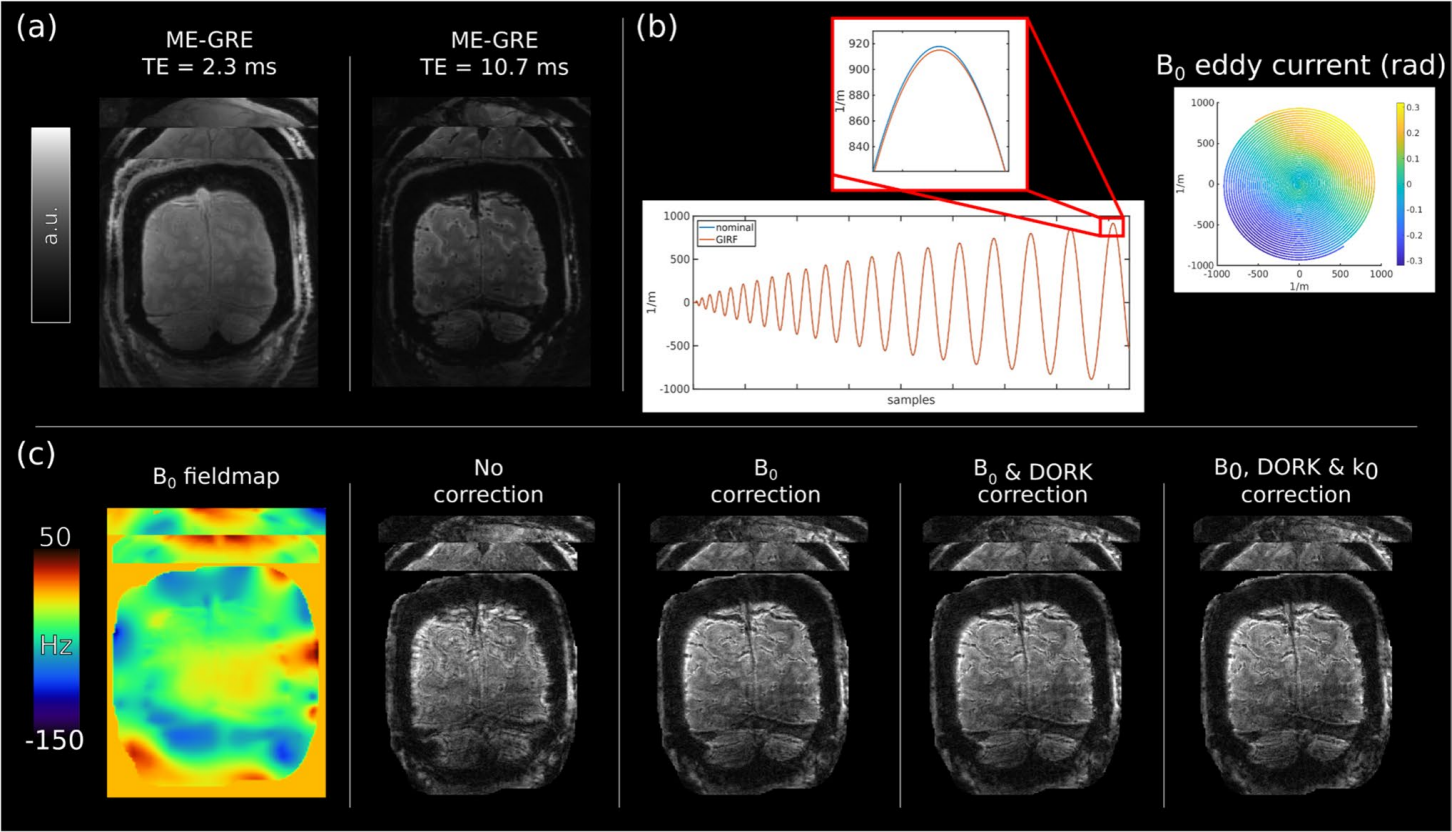
Corrections of spiral data. **a** First and third echo of the low-resolution ME-GRE scan. **b** Nominal versus GIRF predicted trajectory and *B*_0_ eddy current gridded into k-space. Only a small deviation and delay is present in the nominal trajectory. **c** *B*_0_ map and reconstruction without *B*_0_, DORK, and *k*_0_ corrections (eddy current). A considerable improvement in image quality is achieved with the implemented corrections

Figure 4 shows the effect of the static and dynamic *B*_0_ as well as trajectory corrections that we implemented, by example of a volume acquired 10 min into a functional run. Without the proposed corrections, images are of poor quality and unsuitable for fMRI analysis. No significant difference is found between the nominal and GIRF predicted trajectory. The implemented multi-frequency interpolation off-resonance (static) correction significantly improves the image quality. No improvement is achieved when performing DORK (scanner drift) and *K*_0_ (zero-order) corrections, showing that no significant scanner drift and *K*_0_ fluctuations are present during the acquisition. Some remaining *B*_0_ artifacts can be seen especially in areas with high susceptibility differences such as the edge of the brain.

**Fig. 5:**
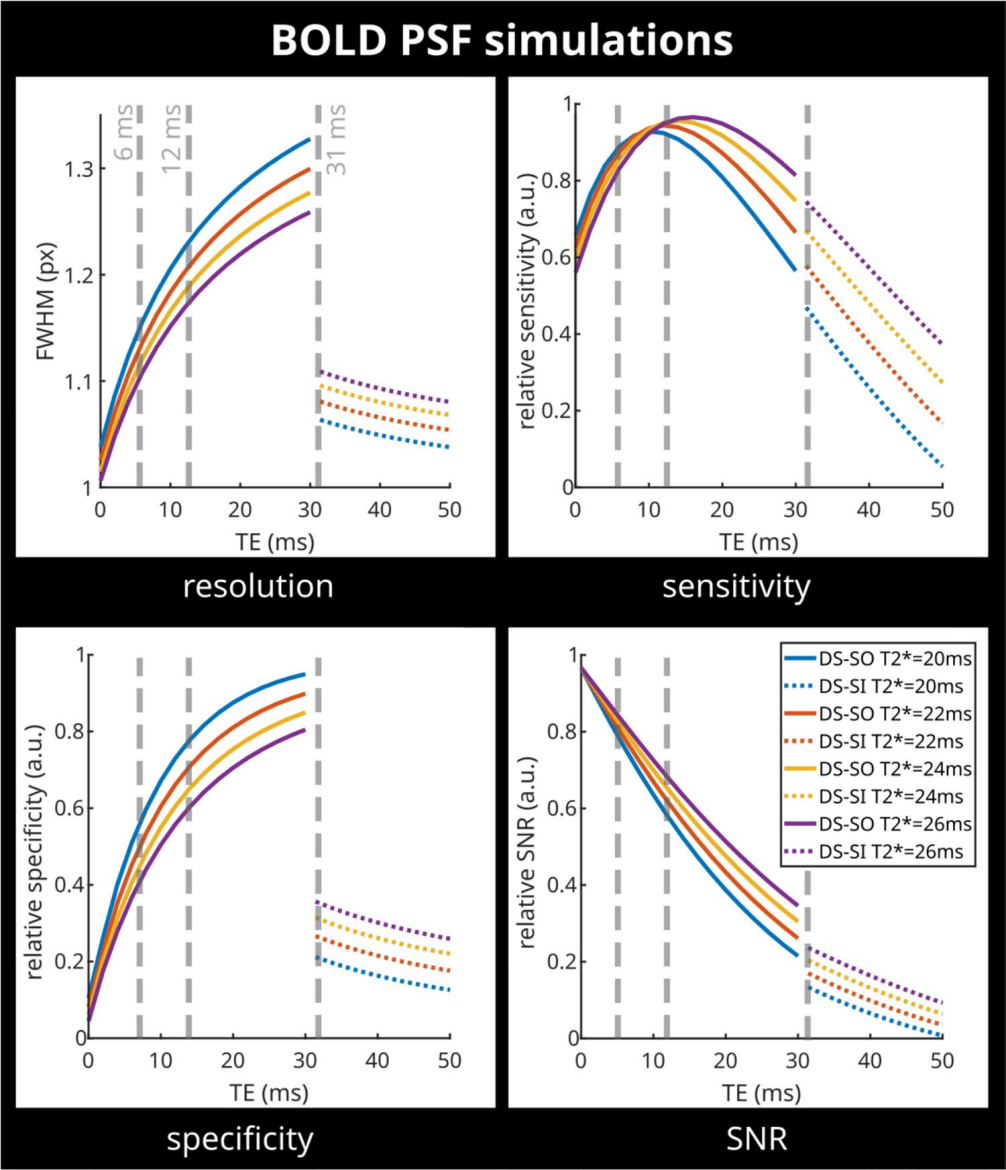
BOLD point spread function simulations for T_2_* = 20–26 ms (values normalized to its maximum) for dual-shot spiral-out (DS-SO) with TE = 2–30 ms and dual-shot spiral-in with TE = 30–50 ms. Dual-shot spiral-out (DS-SO) FWHM increases with TE; the opposite holds for dual-shot spiral-in (DS-SI). DS-SO has the highest sensitivity at TE < T_2_* and DS-SI at the shortest TE. Specificity increases with TE for DS-SO and decreases for DS-SI. SNR is reduced as a function of TE for both DS-SO and DS-SI

Figure 5 shows the results of the BOLD PSF simulations for dual-shot spiral-out (DS-SO) and dual-shot spiral-in (DS-SI) readouts with different echo times for a T_2_* range of 20 to 26 ms. For spiral-out readouts, longer TE increases the FWHM (compromising effective resolution) and specificity while reducing SNR; sensitivity peaks at TE < T_2_*. For spiral-in, longer TE reduces FWHM, sensitivity, specificity, and SNR. Simulations show that TE = 12 ms achieves higher sensitivity than TE = 6 ms for all the different T_2_* values used here. For spiral-in, the shortest possible TE is desired.

**Fig. 6:**
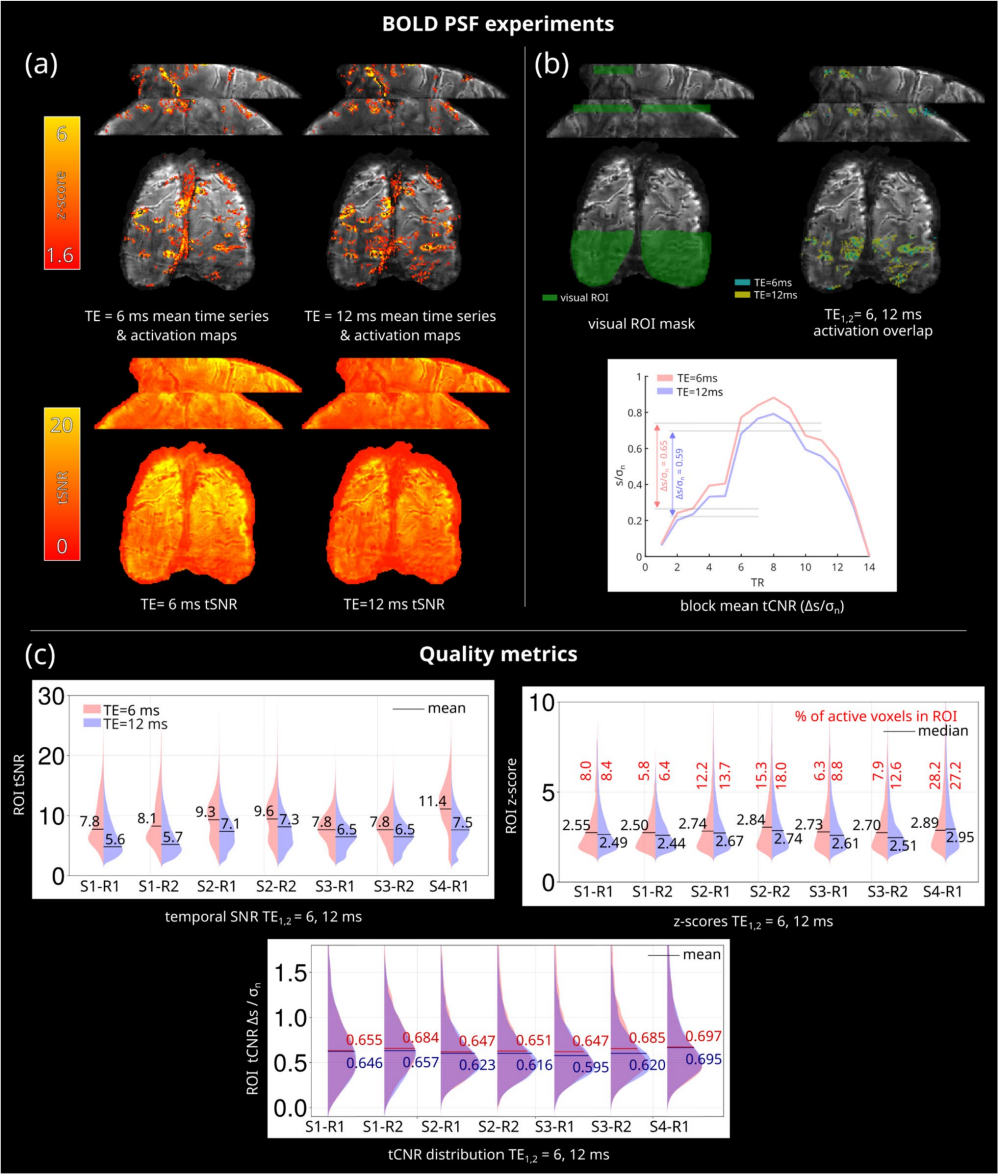
Results of the BOLD PSF experiments. **a** Activation pattern and tSNR for TE = 6 ms and TE = 12 ms acquisitions (S3-R2). Very similar activation patterns are achieved with both TEs, with TE = 6 ms being more prone to inflow effects as seen in the apparent activation on the superior sagittal sinus. As expected, a shorter TE brings an improvement in tSNR. **b** Manually drawn ROI in V1 used for quality metrics (S3-R2), overlapped with binary activation regions for both echo times and block mean tCNR. The activation pattern of both echo times is comparable. **c** ROI tSNR, z scores, and tCNR plots; an average of 33% tSNR improvement and slightly higher median z scores are achieved with the shorter TE. On average, only 1.6% more active voxels are found with the longer TE. The tCNR plot shows that a slightly higher mean tCNR is achieved with TE = 6 ms for all subjects. On average, the tCNR at TE = 6 ms is 4% higher than TE = 12 ms

Results of the BOLD PSF experiments with TE of 6 and 12 ms are shown in Fig. 6. Panel (a) shows results from subject 2; it can be seen that the activation pattern is similar for both echo times. The block mean activation plot in panel (b) shows that the activation pattern is comparable between the two echo times, with a 10% higher tCNR (Δ*s*/*σ*_*n*_) for TE = 6 ms. Panel (c) shows comparison results of tSNR, z scores, and tCNR for all the subjects and runs inside a ROI. An improvement in tSNR and median z score for TE = 6 ms is found for all of the subjects, as expected from the higher SNR at shorter TE. There is a higher percentage of active voxels with TE = 12 ms compared to TE = 6 ms, whereas TE = 6 ms provides a 4% higher average tCNR compared to TE = 12 ms. These results can be explained by the BOLD PSF simulations (Fig. 5) that show a higher sensitivity for TE = 12 ms and higher SNR for TE = 6 ms at different T_2_* values.

**Fig. 7:**
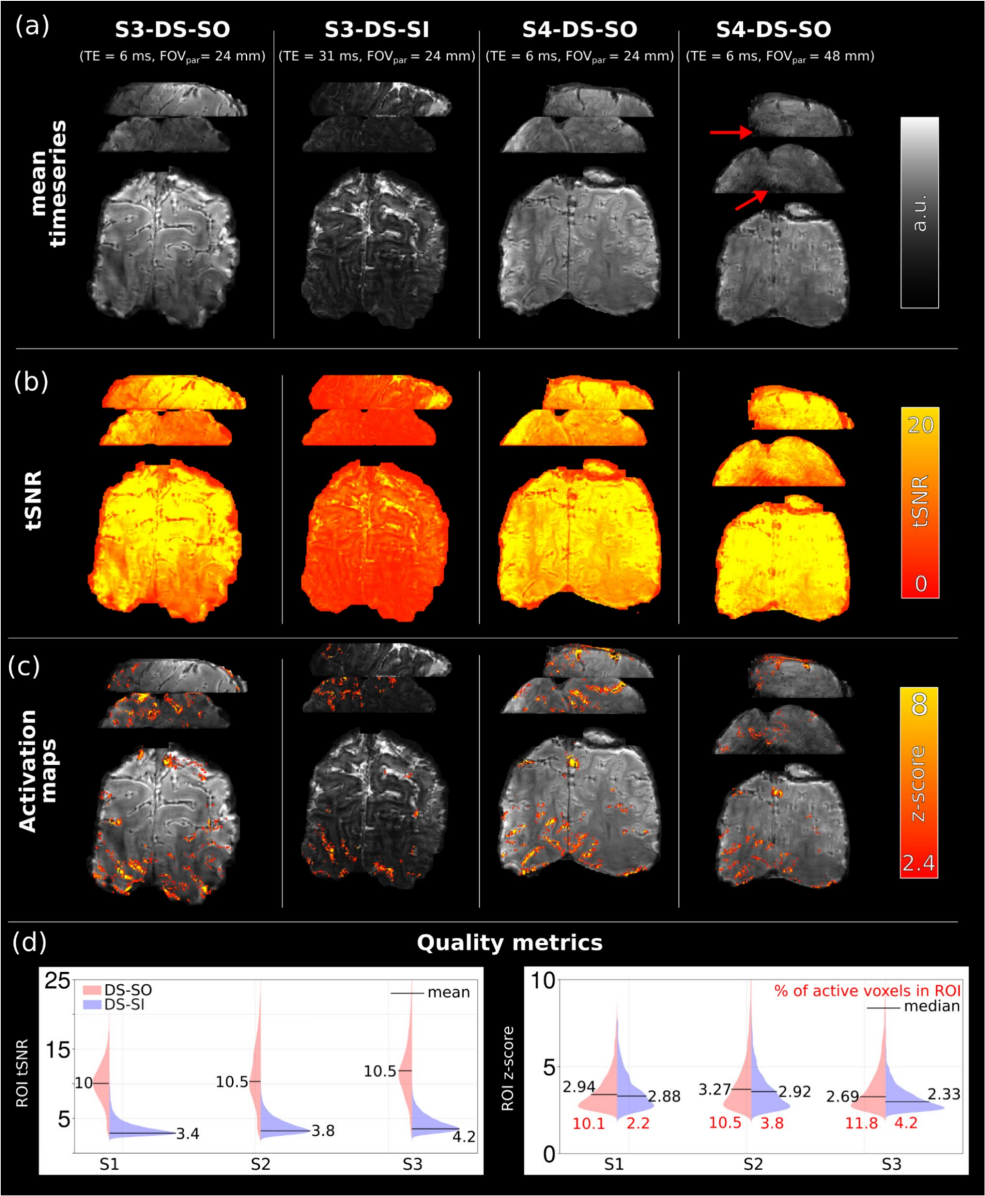
Functional run results for two subjects. DS-SO (FOV_partition_ = 24 mm, *R*_*z*_ = 1), DS-SO (FOV_partition_ = 48 mm, *R*_*z*_ = 2), and DS-SI acquisitions. **a** Mean time series, **b** tSNR, and **c** activation maps. The DS-SO approach has a high tSNR and significant activation, while DS-SI has low tSNR and poor activation detection. Increasing the FOV_partition_ and using an *R*_*z*_ = 2 partition undersampling resulted in good quality images, although some artifacts are present (red arrows). **d** Visual ROI tSNR and *z* scores for the DS-SO and DS-SI acquisitions (FOV_partition_ = 24 mm), DS-SO outperforms DS-SI in all metrics for all subjects

Results from the dual-shot spiral-out and dual-shot spiral-in experiments are shown in Fig. 7 for two different subjects. Mean time series, temporal SNR, and activation maps are shown in panels (a), (b), and (c), respectively. The low tSNR of the DS-SI readout translates into limited detection sensitivity. Increasing the FOV_partition_ by a factor of two and using a partition undersampling factor of two, together with CAIPI-like blips, produces images of good quality, although some artifacts are present in lower slices of the volume and limited activation is found (Fig. 7a red arrows). Panel (d) of Fig. 7 shows the results of the three subjects scanned with both DS-SO and DS-SI readouts with a FOV_partition_ = 24 mm. The DS-SO provides an almost threefold increase in tSNR compared to DS-SI; no significant difference in median z score is found, and an average of three times more active voxels is present with the spiral-out compared to the spiral-in.

## Discussion

In this work, we performed simulations of the BOLD PSF, and functional experiments validated that a TE < T_2_* provides high sensitivity when using dual-shot spiral-out readouts. The use of a shorter TE additionally benefits from increased tSNR and reduced off-resonance effects. Here in our comparison, the TE = 6 ms sequence appears as a suitable compromise with ~33% higher SNR, only marginally reduced activation volume, slightly higher tCNR, and with a ~0.5 s shorter TR_volume_ than for the TE = 12 ms acquisition.

Spiral-*in* readouts, by contrast, were found to be challenging for high-resolution at UHF due to their low tSNR and limited detection sensitivity. We believe, however, that spiral-in readouts can still be beneficial for lower resolution experiments, as shown in Ref. [54], where the readout duration is shorter and a higher tSNR is expected. We also showed the flexibility of the proposed sequence to acquire data by covering a larger slab, using undersampling in the partition direction in a CAIPIRINHA-like fashion. Some residual aliasing artifacts were seen that we attribute to aliasing of areas outside the imaging slab in the head-foot direction due to a tightly chosen in-plane FOV.

The use of segmented spirals was key to achieving good image quality. Using single-shot readouts for this resolution would require a long readout resulting in severe off-resonance artifacts that are difficult to correct, as shown in our previous work on single-shot spiral-out readouts [54] (supplementary Fig. 1). By rotating each plane, we were able to cover a larger k-space area, which helped reduce the undersampling artifacts. In this work, we opted to use conservative undersampling factors, but higher in-plane undersampling should be possible to further reduce the TR. We did not find a significant difference in using the GIRF predicted trajectory versus the nominal one, which shows that the pre-emphasis on this system is near to optimal. We also did not find a significant difference when performing DORK and *K*_0_ corrections, which means that the scanner eddy current compensation is good and that no significant scanner drift is present.

We found larger inflow effects for TE = 6 ms compared to 12 ms as apparent in the activation on the superior sagittal sinus; these effects are consistent with existing literature [31, 73, 74]. In this work, we did not attempt to separate the inflow effects from the BOLD ones, so it can be expected that part of the activation found arises from inflow effects. It has been found that this modulates both the magnitude and temporal information for EPI readouts [75] so that a similar effect can be expected for the stack-of-spirals readout employed here. The small increase in active voxels for the TE = 12 ms case can be expected from the BOLD PSF simulations that show a higher sensitivity than TE = 6 ms for T_2_* = 20–26 ms.

The BOLD PSF model used in this work assumes a mono-exponential signal decay, excluding other sources of signal decay such as *B*_0_ inhomogeneities, so the model is not necessarily applicable for tissue in the whole brain. We relied on approximate literature values for T_2_* and the selected TE values were obtained only from the simulations. It is known that T_2_* varies across brain regions; thus, a single TE will, in any case, not be optimal for all brain areas. A possible solution is the use of a multi-echo readout by which multiple images at different TE and, hence, different BOLD weighting are acquired at a cost of longer TR and acquisition efficiency; the duration of each echo readout and resulting echo time increment, however, makes multi-echo acquisitions at high resolution impractical.

A limitation of spiral fMRI is the slow reconstruction, which has to be performed offline and is computationally demanding. The average time needed to reconstruct one spiral volume was 4 min and used ~100 GB of memory. The long reconstruction times and large memory usage arise from the off-resonance correction that requires multiple NUFFT operations to be applied. The use of coil compression techniques [66] reduced the reconstruction time. For this reason, the current reconstruction framework might not be practical on the MR scanner vendor’s reconstruction server.

The spiral readouts used here made use of the available high-gradient amplitude and slew rate of the AC84-II gradient, which allowed us to reduce the readout duration by 20% compared to what is achievable with more conventional whole-body gradient systems. Higher performance gradients would allow faster readouts, making them more robust against signal loss due to T_2_* decay. Faster readouts make it possible to acquire higher resolution data or cover a larger field of view, yet at a cost of more bandwidth and thus also a penalty in acquisition SNR.

The use of concurrent off-resonance measurements for dynamic correction of field fluctuations and trajectory imperfections to higher order should also lead to further improvements in image quality and temporal stability [76]. In this work, we used a CG-SENSE reconstruction, an alternative approach is subspace reconstruction, which allows for higher undersampling factors. It has been explored for various applications of spiral and other non-Cartesian acquisitions [77, 78]. This type of reconstruction may also be beneficial for fMRI.

## Conclusion

In this work, we characterized the tradeoffs in resolution, sensitivity, specificity, and SNR of using spiral readouts for high-resolution BOLD fMRI at UHF. We have shown that a TE < T_2_* provides high BOLD sensitivity for spiral-out readouts; the shorter TE also provides an improvement in tSNR and a reduction of off-resonance effects. We conclude that spiral-in readouts, however, are challenging for high-resolution studies at UHF due to their low tSNR resulting from long readouts and long TE.

We anticipate that segmented spiral-out readouts could play an important role in BOLD fMRI for high-resolution acquisitions, especially for mesoscopic functional experiments at higher fields (>7T), since T_2_* decreases at higher field strengths and short TEs are required. This type of acquisition could be an important alternative to EPI for very high-resolution (<0.8 mm) fMRI at UHF.

## Supplementary Information

Below is the link to the electronic supplementary material.

Supplementary file1 (DOCX 1168 KB)

## Data Availability

The source code, example sequences, and instruction files will be available on GitHub. We welcome requests for assistance regarding the code.
