# Supplementary material for: BOLD fMRI at 9.4T with 3D stack-of-spirals readouts
Source: MAGMA. 2025 Sep 29;39(2):201–15. doi: 10.1007/s10334-025-01298-4 (PMC13124969; doi:10.1007/s10334-025-01298-4)

# Supplementary material

**Supplementary figure 1:** 0.8 mm isotropic data. (a) mean timeseries, (b) effective tSNR and (c) activation maps. Single-shot spiral-out images present severe off-resonance effects even after correction, dual-shot acquisitions have good image quality. For this resolution, the dual-shot spiral-in acquisition has good tSNR and activation, these results differ from the 0.6 mm isotropic data. Additional details can be found in [54].
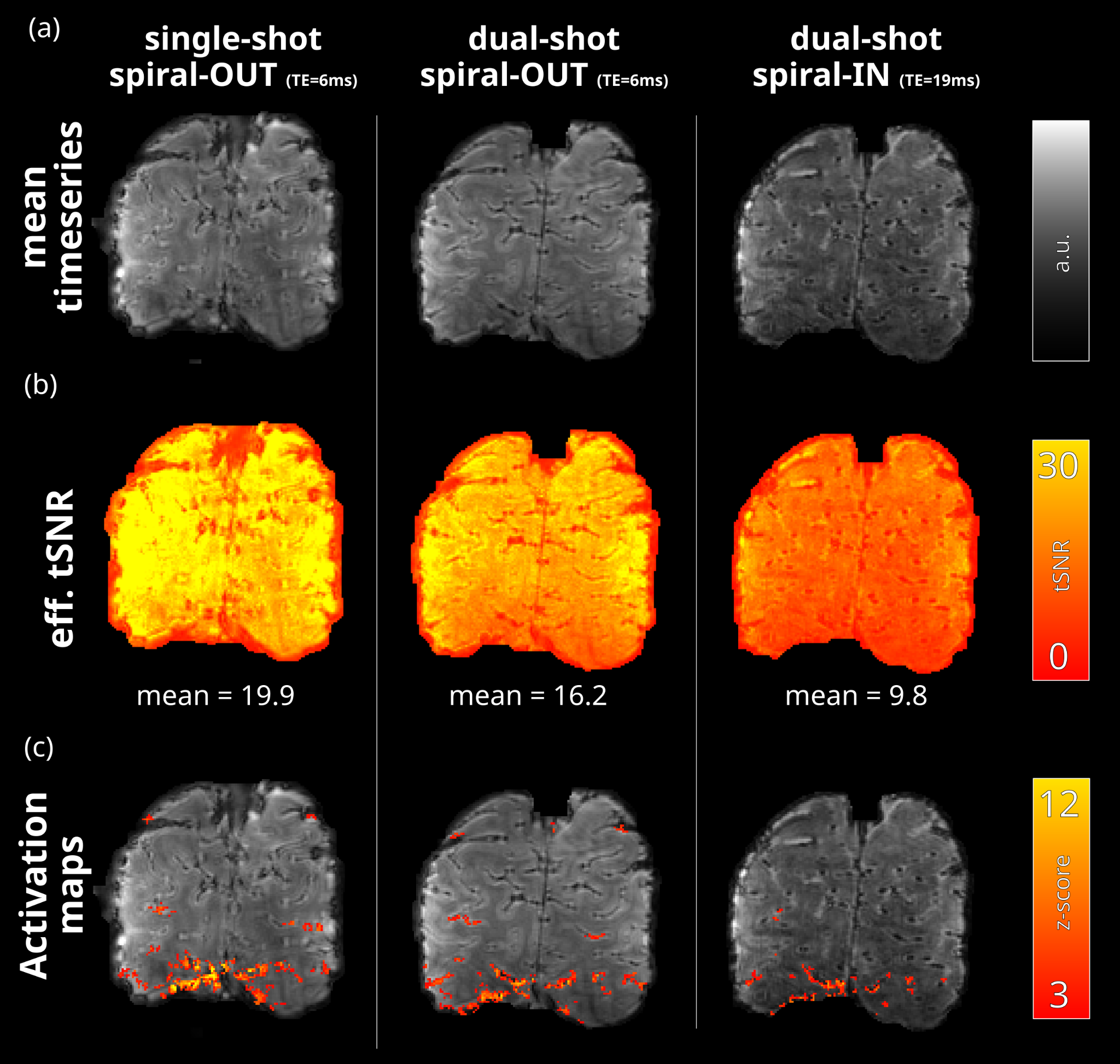

Supplement: Supplementary file 1 — Supplementary file1 (DOCX 1168 KB) [file 10334_2025_1298_MOESM1_ESM.docx]
